# Network Pharmacology-Based Identification of Potential Targets of *Lonicerae japonicae* Flos Acting on Anti-Inflammatory Effects

**DOI:** 10.1155/2021/5507003

**Published:** 2021-09-20

**Authors:** Xiaoying Guo, Xiao Yu, Bingqing Zheng, Longfei Zhang, Fang Zhang, Yongqing Zhang, Jia Li, Gaobin Pu, Lijun Zhang, Haifeng Wu

**Affiliations:** ^1^Shandong University of Traditional Chinese Medicine, Jinan 250300, China; ^2^Shandong Medicine Technician College, Tai'an 271016, China

## Abstract

*Lonicerae japonicae* flos (LJF) is widely used for the treatment of inflammation-related diseases in traditional Chinese medicine (TCM). To clarify the anti-inflammatory mechanism of LJF, 29 compounds with high content in LJF were selected for network pharmacology. Then, a comprehensive network pharmacology strategy was implemented, which involved compound-inflammation-target construction, protein-protein interaction (PPI) network analysis, and enrichment analysis. Finally, molecular docking and *in vitro* experiments were performed to verify the anti-inflammatory activity and targets of the key compound. As a result, 279 inflammation-associated proteins were identified, which are mainly involved in the AGE/RAGE signaling pathway in diabetic complications, the HIF-1 signaling pathway, the PI3K-AKT signaling pathway, and EGFR tyrosine kinase inhibitor resistance. A total of 12 compounds were linked to more than 35 targets, including apigenin, kaempferol, quercetin, luteolin, and ferulic acid. The results of molecular docking showed that AKT has the most binding activity, exhibiting certain binding activity with 10 compounds, including vanillic acid, protocatechuic acid, secologanic acid, quercetin, and luteolin; the results of qRT-PCR and WB confirmed that two key compounds, secologanic acid and luteolin, could significantly decrease the secretion of TNF-*α* and the AKT expression of RAW264.7 murine macrophages stimulated by LPS (lipopolysaccharide). These results demonstrate that the comprehensive strategy can serve as a universal method to illustrate the anti-inflammatory mechanisms of traditional Chinese medicine by identifying the pathways or targets.

## 1. Introduction

Inflammation is the body's protective response to injury or infection, but insufficient or excessive inflammation increases the incidence of many diseases [1]. In the process of the inflammatory response, cytokines play an important bidirectional regulatory role. In the early stage of inflammation, proinflammatory cytokines, such as TNF-*α*, IL-8, IL-1*β*, and IFN-*γ*, are predominant, which can eliminate the threat of infection or trauma through activating a variety of immune cells and promoting the inflammatory response. In the late stage of inflammation, anti-inflammatory cytokines such as IL-10 and IL-13 are dominant, which can weaken and control the inflammatory response [2]. However, in some special cases, the bidirectional regulation pathway of cytokines is damaged, and proinflammatory cytokines continue to play their roles, leading to a large number of immune cells being activated in some parts of the body and even forming inflammatory storms (also known as cytokine storms) in severe cases [3]. Inflammatory storms may be one of the leading causes of severe complications and death in severely affected patients for some acute respiratory infections, such as COVID-19, SARS-COV (SARS), MERS-COV (Middle East respiratory syndrome), and influenza [4–6].

*Lonicerae japonicae* flos (LJF), the dried flower buds of *Lonicerae japonicae* Thunb, also called Japanese honeysuckle or jīn yín huā, possesses the functions of clearing heat, removing toxins, and dispersing wind-heat, which has been widely used in traditional Chinese medicine to treat various diseases such as cough, fever, sore throat, and influenza infection [7]. LJF has significant anti-inflammatory effects, especially for the prevention and treatment of upper respiratory inflammation [8, 9]. LJF contains a variety of compounds, such as phenolic acids, flavonoids, and iridoids [10]. Most of the studies that have investigated the anti-inflammatory substance material of LJF have mainly focused on the single compound or crude extracts [11, 12]; however, reports are scarce pertaining to the complex interactions between the Chinese herb and cellular proteins or the influence of their interactions on the functions and behaviors of the body [13, 14].

**Figure 1:**
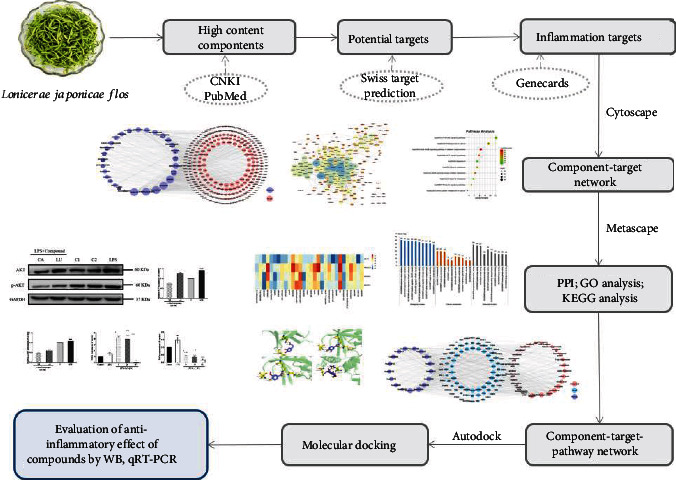
The integrated process of the network pharmacology-based method to identify the anti-inflammatory mechanism of *Lonicerae japonicae* flos.

Network pharmacology is an emerging subject involving the construction of multilayer networks of disease phenotypes, genes, and drugs. Network pharmacology aids in the prediction of new drug targets, deciphering the mode of action, and exploring new drugs [15]. Considering the complexity of the components and functions of TCM, network pharmacology is considered to be an effective approach for identifying key targets and signaling pathways. Ephedra has been widely used to treat asthma in Asia; a strategy of network pharmacology combined with molecular docking and *in vitro* experiments was performed to predict the anti-inflammatory targets of ephedra in treating asthma; the results showed that SELE, IL-2, and CXCL10 are critical targets for ephedra against inflammation due to asthma [16]. In addition, Cui et al. [17] predicted the therapeutic targets of tanshinone I and cryptotanshinone against inflammation and investigated the pharmacological molecular mechanism *in vitro* using a network pharmacology-based strategy. In this study, a comprehensive network pharmacology strategy was carried out, which included the prediction of potential targets, pathway enrichment analysis, and molecular docking. Finally, a murine macrophage cell inflammatory model was built to confirm the predicted result. The entire design of this study is shown in Figure 1.

## 2. Methods

### 2.1. Data Preparation and Construction

The anti-inflammatory chemical components of LJF were gathered from NCBI and PubMed, and a total of 29 components with high content in which were screened for network pharmacology and molecular docking analysis [18–39]. All compound structures were downloaded from the TCMSP (https://tcmspw.com/tcmsp.php) and NCBI PubChem databases (https://pubchem.ncbi.nlm.nih.gov/) and saved in mol2 format. ChemDraw software (v16.0) was used to draw 3D diagrams of the components that were not in the databases.

The online prediction platform SwissTargetPrediction (https://www.swisstargetprediction.ch/) can be used to identify potential targets of natural products and synthetic compounds. The mol2 format files of 29 compounds were imported into the SwissTargetPrediction and TCMSP database, respectively. Then, the prediction targets of the compounds were obtained by integrating the genes collected from the two platforms. The targets were converted into the UniProtKB format using Retrieve/ID mapping (http://www.uniprot.org/uploadlists/) for subsequent enrichment analysis. The GeneCards database (https://www.genecards.org/) was used for collecting inflammation-related targets by imputing in search item “inflammation.” The inflammation targets related to the 29 compounds of LJF were obtained by integrating the compound targets with the inflammation targets and taking the duplicate targets. The compound-target network was constructed using Cytoscape (v3.7.2) software.

### 2.2. Protein-Protein Interaction (PPI) Network

PPI is the process through which two or more proteins form a protein complex through noncovalent bonds [16]. The STRING (v11.0) database (https://string-db.org/) was used to build compound-inflammation target PPI network. “Homo sapiens” was chosen, and a scoring value of >0.9 was selected as the high confidence basis for protein interactions [40]. After eliminating the duplicates, the resultant data were imported into Cytoscape (v3.7.2) for establishing the protein-protein interaction (PPI) network; then, Cytohubba, a plugin of Cytoscape, was used to screen hub genes within the network.

### 2.3. GO and KEGG Pathway Enrichment Analysis

GO is a system widely used for the classification of gene functions and describing the functions of gene products [41]. KEGG pathway enrichment analysis links genomic information to higher-order functional information, suggesting the target that is mainly related to signal pathways. The Metascape database (https://metascape.org/gp/index.html) was used for performing GO and KEGG enrichment analysis. A *P* value of <0.01 is considered statistically significant, and a smaller *P* value indicates a more significant correlation.

### 2.4. Molecular Docking Analysis

Autodock 4.2.0 (http://autodock.scripps.edu/) was used to perform a docking simulation for verifying the credibility of predicted hub genes. All compound structures were downloaded from the ZINC database (http://zinc.docking.org/), and the 3D structures of the targets were downloaded from the RCSB PDB database (http://www.rcsb.org/). The simulation's scoring can be used for evaluating the degree of binding between the compound and the target; the smaller the score, the stronger the binding activity.

### 2.5. Cell Culture

The murine macrophage cell line RAW264.7 was purchased from the Type Culture Collection of the Chinese Academy of Sciences, Shanghai, China. The cells were cultured in DMEM supplemented with 10% FBS at 37°C in a 5% CO_2_ atmosphere.

### 2.6. Drugs and Reagents

Luteolin and secologanic acid were purchased from Shanghai Yuanye Biotechnology Co., Ltd. (Shanghai, China), dimethylsulfoxide (DMSO) and lipopolysaccharide (LPS) were purchased from Sigma (MO, USA), Dulbecco's modified Eagle's medium (DMEM) and trypsin were purchased from Gibco (USA), the BCA assay kit was purchased from SparkJade (Shandong, China), and antibodies were purchased from Wanleibio (China).

### 2.7. MTT Assay

The RAW264.7 cells in the treated groups were pretreated with secologanic acid (5, 10, 20, 40, and 80 *μ*M) or luteolin (5, 10, 20, 40, and 80 *μ*M) for 1 h, followed by treatment with LPS (1 *μ*g/mL, Sigma) for 24 h. The cells in the control group or LPS group were incubated with only medium or LPS (1 *μ*g/mL) for 24 h. After removing the culture solution, the cells were washed with PBS 3 times and incubated with 5 mg/mL MTT (3-(4,5-dimethylthiazol-2-yl)-2,5-diphenyltetrazolium bromide) at 37°C for 4 h. Then, 150 *μ*L of DMSO was added to stop the reaction, and the reaction liquid was detected at 570 nm. Each experiment was repeated 3 times.

### 2.8. Quantitative Real-Time PCR

The RAW264.7 cells in the drug groups were pretreated with caffeic acid (5, 20, and 80 *μ*M) or luteolin (5, 20, and 80 *μ*M) for 1 h, followed by treatment with LPS (1 *μ*g/mL) for 24 h. The cells in the control group or LPS group were incubated with only medium or LPS (1 *μ*g/mL) for 24 h. The total RNA was collected using TRIzol (Invitrogen, CA, USA) reagent; the mRNA was reverse-transcribed to cDNA with a PrimeScript RT Reagent Kit (Tiangen, China); cDNA was mixed with Universal SYBR Green Fast qPCR Mix kits (ABclonal, China) and each primer pair to produce a 20 *μ*L reaction mixture. The levels of *β*-actin were considered to be an endogenous control, and the expansion conditions were as follows: heating to 95°C for 3 min, 45 cycles of 95°C for 5 s, and 60°C for 32 s, and the 2^−*ΔΔ*CT^ method was used to calculate the relative fold changes. The primers are listed as follows: TNF-*α*, forward: 5′-CTCTTCTGCCTGCTGCACTTTG-3′ and reverse: 5′-ATGGGCTACAGGCTTGTCACTC-3′; *β*-actin, forward: 5′-CATTGCTGACAGGATGCAGAAGG-3′ and reverse: 5′-TGCTGGAAGGTGGACAGTGAGG-3′.

### 2.9. Western Blot

Western blotting was performed as reported previously [42]. The RAW264.7 cells in the drug groups were pretreated with secologanic acid or luteolin (20 *μ*M) for 24 h and then treated with LPS (1 *μ*g/mL) for 0.5 h. The cells in the control group or LPS group were separately incubated with medium or LPS (1 *μ*g/mL) for 24 h. The cells were washed with ice-cold PBS and lysed in ice-cold RIPA lysis buffer containing PMSF. Homogenates were centrifuged at 12,000 rpm for 20 min at 4°C, filtered through a Millipore filter with a pore size of 0.45 *μ*m, and stored at −80°C until use. Protein concentrations were quantified by BCA assay. The proteins (20 *μ*g) were resolved by 10% sodium dodecyl sulfate polyacrylamide gel electrophoresis (SDS-PAGE) and transferred onto polyvinylidene difluoride (PVDF) membranes. The membranes were blocked for 2 h at room temperature with 5% nonfat milk/TBST, probed with the appropriate primary antibodies (AKT, p-AKT, and GAPDH) (1 : 1000 dilution), and incubated overnight at 4°C. Then, the membranes were incubated with an HRP-conjugated secondary antibody (1 : 5000 dilution) for 1 h at room temperature, and bands were detected by enhanced chemiluminescence.

### 2.10. Statistical Analysis

Integrated band intensities in the western blot analysis were determined using the ImageJ software. All data are presented as *X* ± SD. Differences between the groups were analyzed by one-way analysis of variance, followed by the least significant difference test using IBM SPSS Statistics (v.25.0) software. A *P* value of <0.05 was considered statistically significant.

## 3. Results and Discussion

### 3.1. Data Preparation and Construction

**Figure 2:**
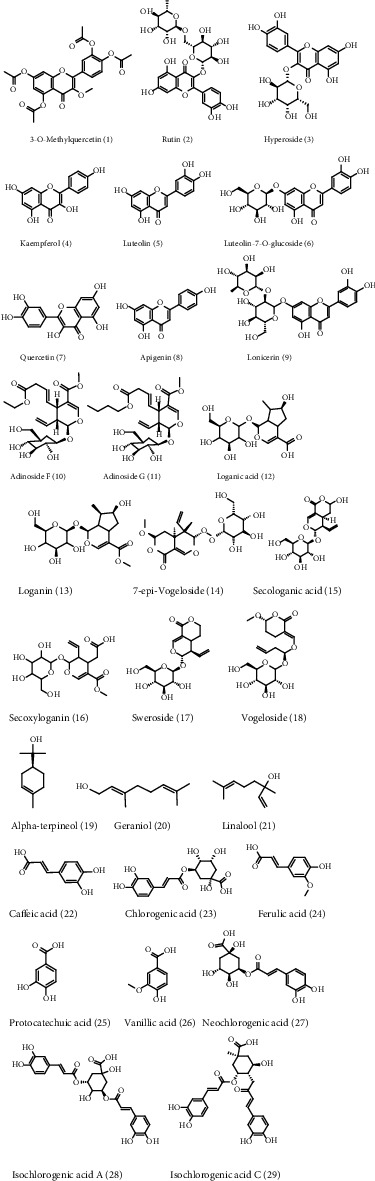
The structure of the 29 active compounds in *Lonicerae japonicae* flos.

**Figure 3:**
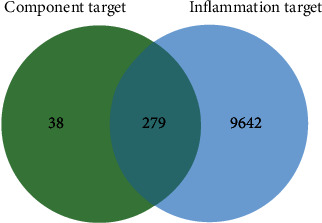
Venn diagram of 317 component targets and 9921 inflammation targets. The 279 inflammatory targets overlap in the middle.

**Figure 4:**
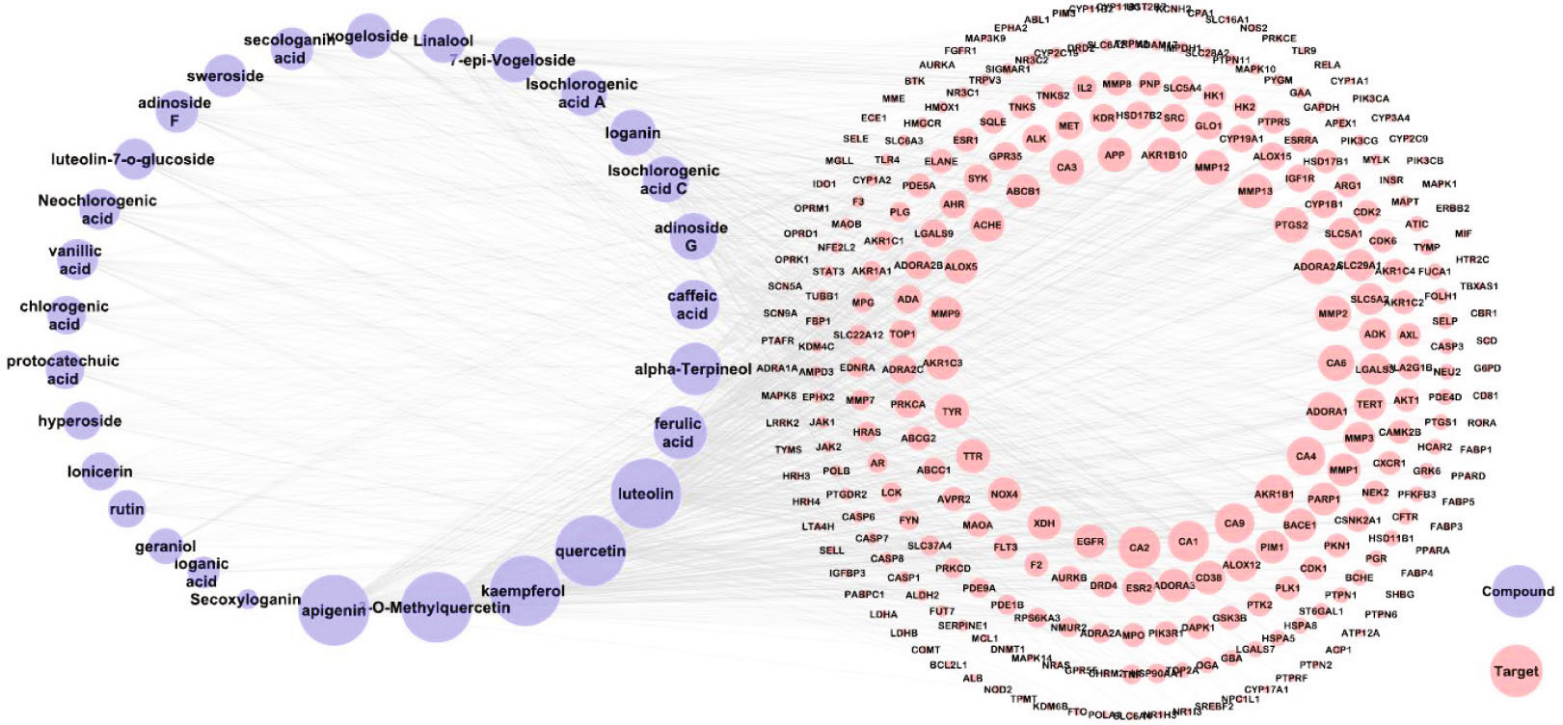
Component-target (C-T) interaction network of *Lonicerae japonicae* flos. The purple ellipses represent the component, the pink nodes represent the target, and the edges represent the relationship between components and the targets. The size of the nodes in the figure is associated with the degree in the network.

More than 217 compounds were isolated and identified from LJF. Among them, 58 compounds have anti-inflammatory effects. A total of 29 compounds (Figure 2) with high content, including flavonoids (compounds 1-9), iridoids (compounds 10-18), volatile oils (compounds 19-21), and organic acids (compounds 22-29), were selected for network pharmacology and molecular docking research. Collectively, 1298 target proteins were retrieved from the SwissTargetPrediction database. After overlapping, 317 protein targets were converted to 317 UniProtKB identifiers by employing ID mapping (http://www.uniprot.org/uploadlists/) for the following enrichment analysis. A total of 279 repeated targets (Figure 3) were selected as LJF inflammation-related targets by comparing the 317 target proteins with the 9921 inflammation-related targets obtained from the GeneCards database. The compound-target (C-T) network was built in Cytoscape (3.7.2) software by converting the 29 compounds (purple ellipses) and the 279 targets (pink circles) to nodes, totaling 308 nodes and 1119 edges (Figure 4). The different sizes of the target nodes indicated the degree of compounds with the common target protein. The larger the degree, the more the number of corresponding targets or the more corresponding components of this target. Compounds 8 (apigenin), 1 (3-O-methylquercetin), 4 (kaempferol), 7 (quercetin), 5 (luteolin), 24 (ferulic acid), 19 (alpha-terpineol), 22 (caffeic acid), 11 (adinoside G), 29 (isochlorogenic acid C), 13 (loganin), and 28 (isochlorogenic acid A) were linked to more than 35 targets and can be considered the main active compounds of LJF (Table 1). Critically, the degree values of 25 compounds were higher than 19, and most of them had anti-inflammatory effects. The degree values of 22 targets, including CA2, CA1, CA9, CA4, and AKR1B1, were higher than 10, indicating that these targets perhaps play a key role in the anti-inflammatory effect for LJF (Table 2). The results of this study show that multiple targets can connect with the same compound, and a single target can interact with multiple compounds, indicating that LJF plays a therapeutic role through the synergy of multiple compounds and targets.

### 3.2. Protein-Protein Interaction (PPI) Network

**Figure 5:**
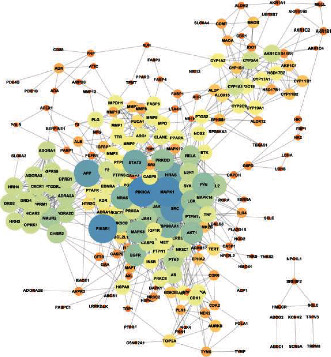
Protein-protein interaction (PPI) network analysis of 224 potential targets. The nodes indicate proteins, and edges represent protein-protein associations. The closer and the larger the nodes are, the higher the degree of freedom they have.

**Figure 6:**
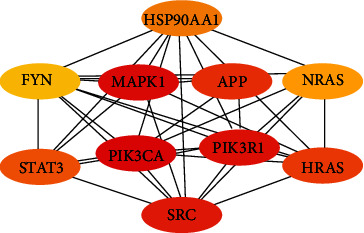
Top 10 genes' interaction network of *Lonicerae japonicae* flos. The nodes indicate proteins, and edges represent protein-protein associations. The depth of the color shade indicates the high degree of the node.

The compound-inflammation target PPI network was constructed by inputting all the 279 LJF inflammation-related targets into the database of STRING and then inputting the PPI network into Cytoscape 3.7.2 software to be visualized. As shown in Figure 5, the PPI network contained 224 functional nodes and edges. The bigger the nodes, the higher the degree of interactivity, indicating a stronger interaction among the proteins. Then, Cytohubba (a plugin of Cytoscape) was used for screening hub genes in the PPI network. As shown in Table 3, the degree values of 20 targets were higher than 23, which indicates that they had more interaction with other targets and might play key roles in treating inflammation. The top 10 targets, including PIK3CA (phosphatidylinositol-4,5-bisphosphate 3-kinase catalytic subunit alpha), MAPK1 (mitogen-activated protein kinase 1), PIK3R1 (phosphoinositide-3-kinase regulatory subunit 1), SRC (SRC proto-oncogene), APP (amyloid beta precursor protein), HRAS (HRas proto-oncogene), STAT3 (signal transducer and activator of transcription 3), HSP90AA1 (heat shock protein 90 alpha family class A member 1), NRAS (NRAS proto-oncogene), and FYN (FYN proto-oncogene), were selected according to their degree values (Figure 6). Among them, target PIK3CA interacted the most with other proteins.

### 3.3. GO (Gene Ontology) Analysis

**Figure 7:**
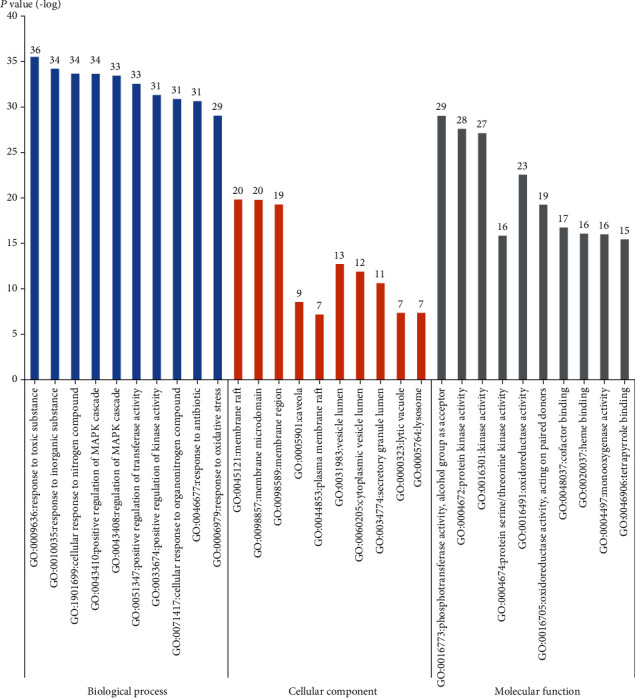
Gene ontology enrichment with top 10 *P* value for each item. The blue columns, the orange columns, and the gray columns are biological process, cellular component, and molecular function, respectively. The *y*-axis stands for the *P* values of fold change.

GO is a widely used system for the classification of gene functions and describing the functions of gene products [41]. GO analysis of the anti-inflammatory targets of LJF was executed for three factors: biological process (BP), molecular function (MF), and cell composition (CC). The *P* values of 1844 biological processes, 41 cell components, and 236 molecular functions were less than 0.01. As shown in Figure 7, the top 10 enrichment results of BP were as follows: response to toxic substance, response to inorganic substance, cellular response to nitrogen compound, positive regulation of MAPK cascade, regulation of MAPK cascade, positive regulation of transferase activity, positive regulation of kinase activity, cellular response to organonitrogen compound, response to antibiotic, and response to oxidative stress. The top 10 enrichment results of MF were phosphotransferase activity (alcohol group as acceptor), protein kinase activity, kinase activity, protein serine/threonine kinase activity, oxidoreductase activity, oxidoreductase activity acting on paired donors with the incorporation or reduction of molecular oxygen, cofactor binding, heme binding, monooxygenase activity, and tetrapyrrole binding.

### 3.4. KEGG Pathway Enrichment Analysis

The inflammatory process can trigger different diseases depending on the specific inflamed tissue or organ involved. However, all disorders have common features or a conjoint cellular process, such as the activation of a stress signaling pathway and the concomitant production of inflammatory cytokines [43]. A total of 279 LJF inflammation-related targets were input to the Metascape database, and 173 pathways that had statistical significance were input for pathway enrichment analysis. The 10 pathways with the lowest *P* values are documented in Table 4 and include the following: the AGE-RAGE signaling pathway in diabetic complications, the HIF-1 signaling pathway, the EGFR tyrosine kinase inhibitor resistance, the proteoglycans in cancer, the insulin resistance, the prolactin signaling pathway, the PI3K-AKT signaling pathway, the endocrine resistance, the hepatitis B, and central carbon metabolism in cancer.

**Figure 8:**
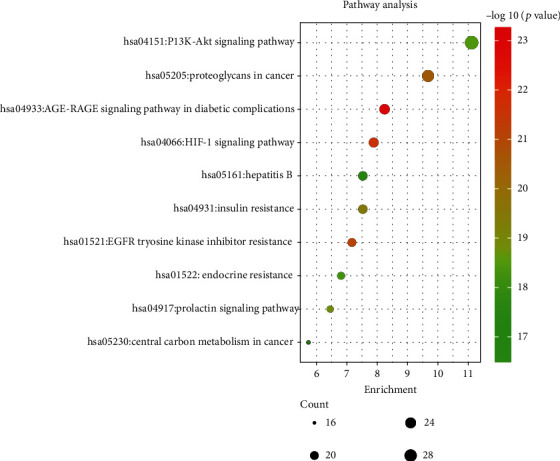
KEGG pathway enrichment with the top 10 *P* value. The *y*-axis stands for enriched pathways of the targets. The color of the bubble is associated with the *P* value, and the size is related to the enrichment number of targets.

**Figure 9:**
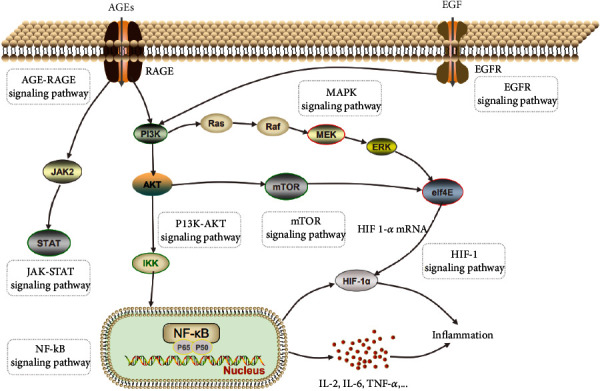
Pharmacological mechanism cascade pathway of *Lonicerae japonicae* flos impact on inflammation.

The top three pathways containing the most LJF inflammation-related targets were as follows: the PI3K-AKT signaling pathway, the AGE-RAGE signaling pathway in diabetic complications, and the HIF-1 signaling pathway (Figure 8), which indicates that the three pathways play a crucial role in the anti-inflammation properties of LJF. The cascade pathway of pharmacological mechanism of LJF acting on inflammation is illustrated in Figure 9.

AKT is a key downstream signaling protein of PI3K. After PI3K is activated, it binds to AKT in the plasma membrane. Then, the activated AKT can promote the expression and secretion of proinflammatory cytokines by activating the NF-*κ*B pathway, resulting in an imbalance of cytokine secretion and a series of inflammatory reactions [44–46]. Several studies have shown that the inhibition of the PI3K signal can inhibit the secretion of proinflammatory factors in macrophages and dendritic cells and increase the secretion of anti-inflammatory factor IL-10 when a Toll-like receptor- (TLR-) mediated inflammatory response occurs [47–52]. The important role of PI3K/AKT/NF-*κ*B signaling pathways in the anti-inflammatory effects of LJF was confirmed in a previous report [53].

In the AGE-RAGE signaling pathway in diabetic complications, highly abundant AGEs in the diabetic milieu of the kidneys upregulate RAGE expression, and ligand-evoked RAGE stimulation leads to the activation of intracellular signaling pathways, including JAK/STAT, MAPK/ERK, PI3K/AKT/mTOR, and NF-*κ*B, of which the common end is the activation of nuclear transcription factors involved in the inflammatory and fibrotic processes [54].

Crosstalk between HIF-1*α* and NF-*κ*B regulates essential inflammatory functions in myeloid cells. HIF-1*α* increases macrophage aggregation, invasion, and motility and drives the expression of proinflammatory cytokines, such as TNR-*α* and IL-6 [55].

**Figure 10:**
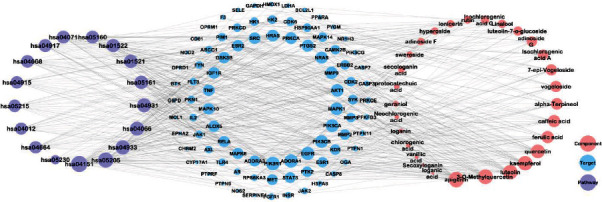
Component-target-pathway (C-T-P) interaction network. The purple, pink, and blue nodes are the pathway, the component, and the target, respectively. The edges represent the relationship between pathway, component, and target. The size of the nodes in the figure is associated with the degree in the network.

The 17 pathways with *P* values of less than 3 × 10^−15^ were selected, and the compound-target-pathway (C-T-P) network was constructed using Cytoscape software (Figure 10). In a network, nodes with high degree values indicate high interconnectedness [56]. The targets AKT1, PIK3CA, PIK3CB, and PIK3R1 were related to all 17 pathways; thus, they were recognized as key targets. Target MAPK connected to 16 pathways simultaneously and had more interactions with other targets in the PPI network. The top five components with higher degree values, including compounds 8 (apigenin), 7 (quercetin), 4 (kaempferol), 1 (3-O-methylquercetin), and 5 (luteolin), were recognized as the key components, with degree values of 90, 89, 89, 89, and 88, respectively, indicating that these components are correlated with at least 88 of the 90 targets within the 17 pathways. In addition, another seven components, including compounds 24 (ferulic acid), 19 (*α*-terpineol), 22 (caffeic acid), 11 (adinoside G), 18 (vogeloside), 14 (7-epi-vogeloside), and 13 (loganin), had degree values higher than 30, which suggests that they also play important roles in the anti-inflammation effects of LJF.

### 3.5. Molecular Docking Analysis

**Figure 11:**
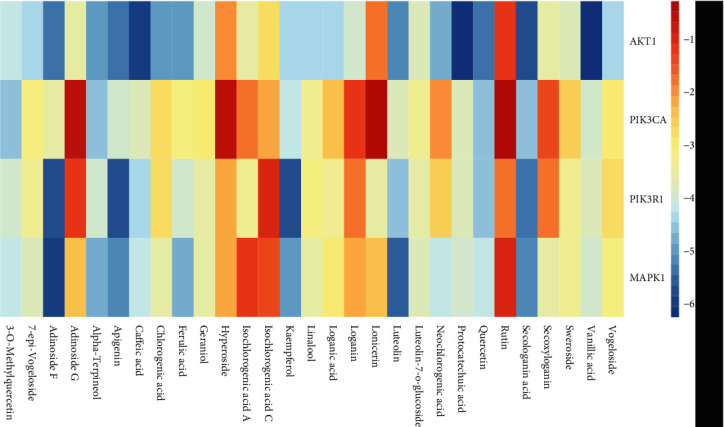
Heat maps show docking scores of hub genes combining to 29 components of *Lonicerae japonicae* flos. Color represents binding energy score.

**Figure 12:**
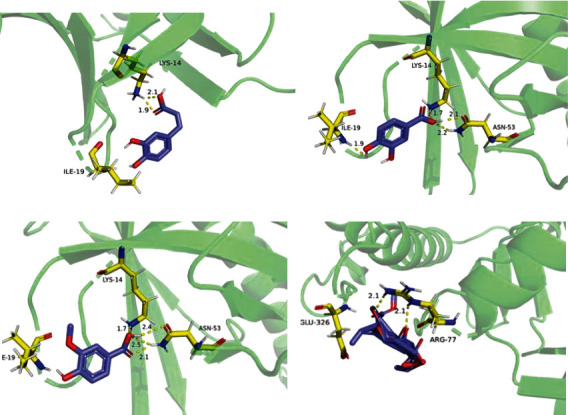
Component-target docking combination: (a) caffeic acid-AKT1 (score -6.11); (b) protocatechuic acid-AKT1 (score -6.39); (c) vanillic acid-AKT1 (score -6.52); (d) adinoside F-MAPK1 (score -6.01).

The dock results of 29 components and hub genes (AKT, PIK3R1, PIK3CA, and MAPK1) are shown in Figure 11, and the docking ligand-protein binding energy is summarized in Table 5. It is generally considered that the value of the Autodock Vina score indicates the binding activity between a compound and a protein [16]. Molecular docking was used for the verification of the interactions between ingredients and target genes [57]. Among the four targets, AKT has the most binding activity, which exhibited binding activity with 10 compounds, and their Vina scores were all less than -5. These 10 compounds are as follows: vanillic acid, protocatechuic acid, caffeic acid, secologanic acid, quercetin, apigenin, adinoside F, luteolin, ferulic acid, and alpha-terpineol. In addition, there were four components, including secologanic acid, adinoside F, apigenin, and kaempferol, which exhibited binding activity (binding energy ≤ −5) with the targets PIK3R1 and MAPK1. In addition, luteolin and target MAPK1 also had binding activity. The top four compound-target complexes were screened by sorting the docking scores in descending order, as shown in Figure 12. Compounds 26 (vanillic acid), 25 (protocatechuic acid), and 22 (caffeic acid) exhibited binding activity with the target AKT1, and compound 10 (adinoside F) showed binding activity to the target MAPK1. Previous studies showed that protocatechuic acid may inhibit the LPS-stimulated inflammatory mediator production in keratinocytes by reducing the Toll-like receptor-4-dependent activation of AKT, mTOR, and NF-*κ*B pathways and the activation of JNK and p38-MAPK [58]. Caffeic acid inhibited the inflammatory response by downregulating the phosphorylation of several important transcriptional factors, such as NF-*κ*B and STAT-3 [59]. In addition, vanillic acid inhibited the LPS-induced production of tumor necrosis factor TNF-*α* and interleukin IL-6 by suppressing the activation of NF-*κ*B and caspase-1 [60].

### 3.6. The Appropriate Concentrations of Luteolin and Secologanic Acid

**Figure 13:**
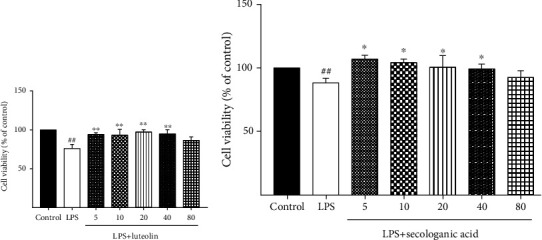
Effect of different concentrations of luteolin and secologanic acid on RAW264.7 macrophage viability. (a) RAW264.7 cells were incubated with luteolin (5, 10, 20, 40, and 80 *μ*M) for 24 hours after being treated with 1 *μ*g/mL LPS. (b) RAW264.7 cells were incubated with secologanic acid (5, 10, 20, 40, and 80 *μ*M) for 24 hours after being treated with 1 *μ*g/mL LPS. Data are expressed as the mean ± SD of three independent experiments. ^##^*P* < 0.01 compared with the control group; ^∗^*P* < 0.05 and ^∗∗^*P* < 0.01 compared with the model group.

According to the results of the compound-target-inflammation network (Figure 10) analysis, secologanic acid and luteolin are the main active compounds of LJF. MTT was performed to detect the viability of RAW264.7 cells, and the suitable concentrations of luteolin and secologanic acid in treating RAW264.7 cells were obtained.  Figure 13 shows that treatment with various concentrations of luteolin (5, 10, 20, 40, and 80 *μ*M) and secologanic acid (5, 10, 20, 40, and 80 *μ*M) for 24 h had no cytotoxicity on the vitality of RAW264.7 cells. Therefore, concentrations of 5, 20, and 80 *μ*M for luteolin and secologanic acid were selected as the treating concentrations in this study.

### 3.7. Luteolin and Secologanic Acid Inhibit the Production of TNF-*α*

**Figure 14:**
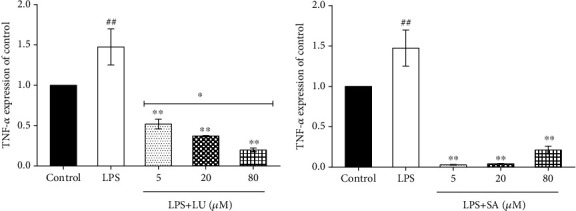
Effect of luteolin and secologanic acid on the mRNA levels of TNF-*α*. TNF-*α* levels of RAW264.7 were analyzed by qRT-PCR after incubation with LPS by 24 h. LU: luteolin; SA: secologanic acid. ^##^*P* < 0.01 versus the control group. ^∗∗^*P* < 0.01 versus the LPS group. ^∗^Above the horizontal line *P* < 0.05 versus other dose groups. The data were represented as the mean ± SD of three independent experiments.

Tumor necrosis factor-alpha (TNF-*α*) is one of the major mediators of inflammation. Induced by a wide range of pathogenic stimuli, TNF-*α* induces other inflammatory mediators and proteases that orchestrate inflammatory responses. The proinflammatory effects of TNF-*α* are primarily due to its ability to activate NF-*κ*B. Studies showed that downregulating the expression of TNF-*α* could inhibit inflammatory reactions, improve cardiac function, and inhibit oxidative stress [61]. To further confirm the anti-inflammatory activity of secologanic acid and luteolin, the levels of TNF-*α* in LPS-induced RAW264.7 cells were detected. As shown in Figure 14, LPS significantly increased the secretion of TNF-*α* compared to the control group. Luteolin, at 5, 20, and 80 *μ*M, significantly inhibited the generation of TNF-*α* in a dose-dependent manner. Similarly, the expression of TNF-*α* in the 5, 20, and 80 *μ*M secologanic acid-treated groups also significantly decreased compared to the LPS groups, but not in a dose-dependent manner.

### 3.8. Luteolin and Secologanic Acid Downregulated the Expression of AKT

**Figure 15:**
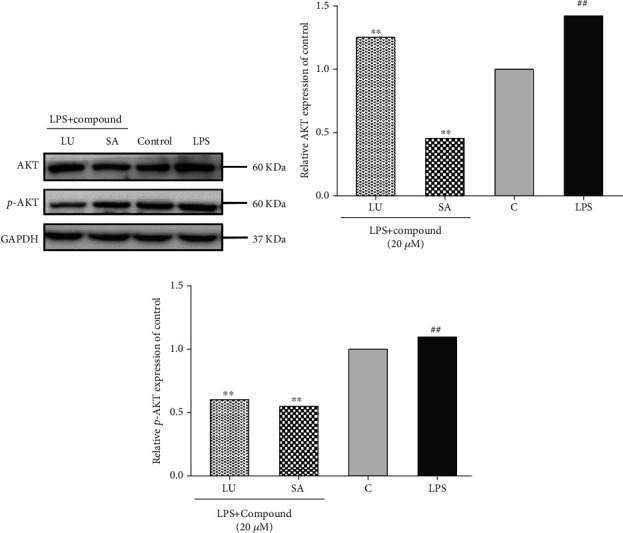
Effect of luteolin and secologanic acid on the expression of AKT and p-AKT in LPS-induced RAW264.7. (a) AKT and p-AKT levels of RAW264.7 were analyzed by western blotting after incubation with LPS for 0.5 h. (b) Relative AKT expression of control. (c) Relative p-AKT expression of control. LU: luteolin; SA: secologanic acid; C: control. ^##^*P* < 0.01 versus untreated macrophage control. ^∗∗^*P* < 0.01 versus the LPS group.

AKT is a subset of the AGC protein Ser/Thr kinase family and plays an important role in cell growth, metabolic regulation, cancer, and other diseases [62]. In the process of inducing inflammation, AKT can be activated through phosphorylation; mediate the activation of downstream NF-KB, mTOR, and other signaling pathways; promote the secretion of proinflammatory cytokine TNF-a; and aggravate inflammation [44]. Studies found that the flavonoid extracts of LJF can reduce the expression level of p-AKT, participate in the PI3K/AKT signaling pathway, and inhibit the secretion of proinflammatory factors [53]. The PPI network and KEGG results all show that LJF's anti-inflammatory functions are involved with AKT, which was further confirmed by molecular docking technology. To further verify the role of AKT, the protein levels of AKT and p-AKT in RAW264.7 macrophagocytes treated with secologanic acid (20 *μ*M) or luteolin (20 *μ*M), separately, were determined *in vitro*. As shown in Figure 15, luteolin and secologanic acid significantly inhibited the relative expression of AKT and p-AKT compared to the LPS treatment (*P* < 0.01). Among them, secologanic acid showed better inhibition effects on the expression and activation of AKT than luteolin, which is consistent with the result of AKT binding (the Vina score of secologanic acid and luteolin with AKT was -5.98 and -5.37, respectively) in molecular docking. This study reveals that network pharmacology and molecular docking are powerful approaches for searching active compounds and hub targets for TCM.

## 4. Conclusions

In this study, the key anti-inflammation compounds and targets of LJF were screened using a network pharmacology strategy and then verified by molecular docking and *in vitro* experiments. The main results are as follows. First, 279 targets were selected as LJF inflammation-related targets by comparing compound-related targets with inflammation-related targets. Second, the network analysis of compounds and targets shows that 12 compounds are linked to more than 35 targets, including apigenin, 3-O-methylquercetin, kaempferol, quercetin, luteolin, ferulic acid, alpha-terpineol, caffeic acid, adinoside G, isochlorogenic acid C, loganin, and isochlorogenic acid A. Third, hub genes, such as PIK3CA, MAPK1, PIK3R1, AKT1, SRC, APP, HRAS, and STAT3, were screened from the protein-protein interaction network. Fourth, the bioactivity of 279 inflammation targets spreads widely, involving response to toxic substance, response to inorganic substance, cellular response to nitrogen compound, positive regulation of MAPK cascade, positive regulation of kinase activity, cellular response to organonitrogen compound, response to antibiotic, and response to oxidative stress. Through building and analyzing the C-T-P network, the top four pathways, including the PI3K-AKT signaling pathway, the AGE-RAGE signaling pathway in diabetic complications, the EGFR tyrosine kinase inhibitor resistance, and the HIF-1 signaling pathway, are assumed to play a crucial role in the anti-inflammation effects of LJF. Fifth, the results of molecular docking show that 10 compounds, including vanillic acid, protocatechuic acid, caffeic acid, secologanic acid, quercetin, apigenin, adinoside F, luteolin, ferulic acid, and alpha-terpineol, exhibited binding activity with target AKT1. Finally, the anti-inflammatory effects and anti-inflammatory mechanisms of two key components, including secologanic acid and luteolin, were tested *in vitro* for further confirming the network pharmacological screening results. The results of qRT-PCR show that both secologanic acid and luteolin can inhibit TNF-*α* generation at the mRNA level. The western blotting results show that the expression levels of AKT and p-AKT were significantly decreased after secologanic acid and luteolin treatment. Secologanic acid showed better inhibition effects than luteolin on the activation of AKT, which is consistent with the results of AKT binding in molecular docking. Notably, secologanic acid is the most abundant iridoid glycoside in LJF [7]. In this study, the anti-inflammatory activity and mechanism of secologanic acid were verified by molecular docking and *in vitro* experiments for the first time.

In conclusion, the results of our study suggest that network pharmacology is a powerful tool for discovering the active compounds and mechanisms of action in TCM.

## Figures and Tables

**Table 1:** 29 components of *Lonicerae japonicae* flos corresponding to the inflammation targets.

No.	Component	Target	Counts
8	Apigenin	NOX4, AKR1B1, XDH, MAOA, FLT3, CYP19A1, ESR1, ACHE, ADORA1, PTGS2, ESR2, CDK6, ADORA2A, SYK, GSK3B, ABCC1, HSD17B1, TTR, CSNK2A1, CFTR, CYP1B1, ABCG2, AKR1B10, TNKS2, TNKS, ALOX5, PARP1, CA2, ABCB1, ALOX12, CA4, PTPRS, GLO1, APP, MMP9, MMP2, MMP12, CD38, TOP1, ARG1, ESRRA, PFKFB3, GRK6, ALOX15, TYR, HSD17B2, AHR, CA1, CA9, CBR1, AR, TERT, PIM1, EGFR, CDK1, LCK, AURKB, TBXAS1, IGF1R, KDR, PLK1, MET, ALK, AXL, BCHE, ADORA3, CDK2, HTR2C, GPR35, DAPK1, MPG, SLC22A12, F2, ST6GAL1, PLG, AVPR2, DRD4, MPO, PIK3R1, SRC, PTK2, MMP13, MMP3, CA3, CA6, PKN1, NEK2, CXCR1, CAMK2B, AKT1	90
1	3-O-Methylquercetin	NOX4, CYP1B1, APP, AKR1B1, XDH, MCL1, CA2, CA4, ABCG2, ABCC1, PLG, ABCB1, IGF1R, EGFR, ADORA1, ACHE, ALOX15, ALOX12, AVPR2, MAOA, FLT3, CYP19A1, F2, PIM1, ALOX5, AURKB, DRD4, GLO1, MPO, PIK3R1, ADORA2A, DAPK1, CA1, GSK3B, SRC, PTK2, HSD17B2, KDR, MMP13, MMP3, CA3, PLK1, CA6, CDK1, MMP9, MMP2, PKN1, CA9, CSNK2A1, MET, NEK2, CXCR1, CAMK2B, ALK, AKT1, PLA2G1B, BACE1, AXL, AKR1C2, AKR1C1, AKR1C3, AKR1C4, AKR1A1, GPR35, CDK6, CDK2, ARG1, SYK, MAPT, TOP2A, INSR, MYLK, PIK3CG, APEX1, TYR, HSD17B1, AHR, ESRRA, TERT, PTPRS, ESR2, MPG, SLC22A12, ADORA3, PARP1, TTR, MMP12, CD38, AKR1B10	89
4	Kaempferol	NOX4, AKR1B1, XDH, TYR, FLT3, CA2, ALOX5, HSD17B2, ABCC1, HSD17B1, AHR, ESRRA, ABCB1, CYP1B1, ABCG2, ADORA1, CA4, ACHE, MAOA, GLO1, SYK, GSK3B, MMP9, MMP2, ALOX15, ALOX12, PTPRS, ADORA2A, ARG1, GPR35, ESR2, DAPK1, MPG, SLC22A12, CDK6, CDK2, TTR, AKR1B10, TNKS2, TNKS, CYP19A1, CSNK2A1, EGFR, AVPR2, IGF1R, F2, PIM1, AURKB, DRD4, MPO, PIK3R1, CA1, SRC, PTK2, KDR, MMP13, MMP3, CA3, PLK1, CA6, CDK1, PKN1, CA9, MET, NEK2, CXCR1, CAMK2B, ALK, AKT1, PLA2G1B, BACE1, AXL, AKR1C2, AKR1C1, AKR1C3, AKR1C4, AKR1A1, APP, PARP1, MMP12, CD38, TOP1, ESR1, PTGS2, CFTR, PFKFB3, GRK6, TERT, BCHE	89
7	Quercetin	NOX4, AVPR2, AKR1B1, XDH, MAOA, IGF1R, FLT3, CYP19A1, EGFR, F2, CA2, PIM1, ALOX5, AURKB, DRD4, ADORA1, GLO1, MPO, PIK3R1, ADORA2A, DAPK1, CA1, GSK3B, SRC, PTK2, HSD17B2, KDR, MMP13, MMP3, CA3, ALOX15, ABCC1, PLK1, CA6, CDK1, MMP9, MMP2, PKN1, CA9, CSNK2A1, ALOX12, MET, CA4, NEK2, CXCR1, CAMK2B, ALK, AKT1, ABCB1, PLA2G1B, BACE1, CYP1B1, AXL, ABCG2, AKR1C2, AKR1C1, AKR1C3, AKR1C4, AKR1A1, GPR35, SYK, MAPT, TOP2A, INSR, ACHE, MYLK, PIK3CG, APEX1, ARG1, PTPRS, ESR2, MPG, SLC22A12, CDK6, CDK2, TYR, HSD17B1, AHR, ESRRA, APP, PARP1, TTR, MMP12, CD38, AKR1B10, TNKS2, TNKS, TOP1, TERT	89
5	Luteolin	NOX4, AKR1B1, XDH, MAOA, FLT3, CA2, ALOX5, ADORA1, GLO1, APP, SYK, GSK3B, PARP1, TTR, MMP9, MMP2, CA4, MMP12, CD38, CYP1B1, ABCG2, AKR1B10, TNKS2, TNKS, TOP1, ARG1, PTPRS, ABCC1, HSD17B1, ACHE, CDK6, ABCB1, HSD17B2, ALOX15, ALOX12, ESR2, CYP19A1, ADORA2A, CSNK2A1, ESR1, PTGS2, CFTR, GRK6, CDK2, TERT, CA1, CA9, CDK1, TYR, AHR, ESRRA, GPR35, DAPK1, AVPR2, IGF1R, EGFR, F2, PIM1, AURKB, DRD4, MPO, PIK3R1, SRC, PTK2, KDR, MMP13, MMP3, CA3, PLK1, CA6, PKN1, MET, NEK2, CXCR1, CAMK2B, ALK, AKT1, PLA2G1B, BACE1, AXL, AKR1C2, AKR1C1, AKR1C3, AKR1C4, AKR1A1, PFKFB3, PLG, AR	88
24	Ferulic acid	CA2, CA1, CA6, CA9, MAOB, ALOX5, MMP9, MMP1, MMP2, PTPN1, CA3, AKR1B1, APP, NFE2L2, STAT3, HSD11B1, ESR2, CA4, TLR4, MET, CYP1A1, CYP1A2, CYP1B1, PTGS1, EGFR, TTR, PTGS2, TUBB1, RELA, ADORA1, ADORA2A, ADORA2B, TLR9, AKR1B10, ALOX15, PRKCE, F3, NOS2, FYN, LCK, SLC16A1, ABCB1, TOP2A, FBP1, BACE1, GLO1, CPA1, KDM4C, AHR, AMPD3, PARP1	51
19	Alpha-terpineol	AR, CYP19A1, CA2, CA1, CA4, CHRM2, SLC6A4, TRPM8, NR1H3, PTPN1, NR1I3, SREBF2, NPC1L1, BCHE, ACHE, SQLE, ESR1, SLC6A2, DRD2, ESR2, CYP17A1, CYP2C19, NR3C2, PTPRF, PTPN2, PLA2G1B, ACP1, AKR1B10, SIGMAR1, TRPV3, NR3C1, ATP12A, PTPN6, SHBG, FABP4, PPARA, FABP3, FABP5, PPARD, FABP1, RORA, HMOX1, HMGCR, PGR, CD81, G6PD, SCD, ADRA2C, HSD11B1, SLC6A3	50
22	Caffeic acid	CA2, ALOX5, CA1, CA6, MMP9, MMP1, MMP2, PTPN1, CA9, CA3, AKR1B1, ESR2, CA4, AKR1B10, HCAR2, MIF, TLR4, ERBB2, ESR1, SLC6A2, TTR, MAPK1, AKR1C3, AKR1C4, AKR1C2, SYK, APP, EGFR, FYN, LCK, PTGS1, PIK3CB, CYP1A2, CYP2C9, CYP3A4, CYP2C19, PIK3CA, ELANE, F3, HSD11B1, MAOB, NFE2L2, STAT3	43
11	Adinoside G	ADORA2A, ADORA1, ADORA3, SLC29A1, MMP3, MMP9, ADAM17, ADORA2B, ADK, ST6GAL1, SLC5A2, MAPK14, SLC5A1, TOP1, IMPDH1, LGALS3, LGALS7, PARP1, TNKS2, TNKS, HSPA5, MMP1, LGALS9, MMP2, CA2, CA1, CA9, OGA, NRAS, PTGS2, MMP13, MMP7, MMP12, MMP8, GPR55, GBA, HK2, HK1	38
29	Isochlorogenic acid C	AKR1B1, APP, AKR1B10, MMP12, MMP2, MMP13, PRKCD, CA4, PRKCA, CA6, CA2, CA1, ABCB1, SLC37A4, CA9, FYN, TTR, MMP1, PDE5A, ELANE, SELE, SELP, PDE4D, PDE9A, PDE1B, HCAR2, PTGDR2, CASP3, MGLL, PIM1, FOLH1, CASP6, CASP7, CASP8, CASP1, EDNRA	36
13	Loganin	TYR, ADORA1, CA2, CA1, CA9, HSP90AA1, ADORA3, ADORA2A, EPHX2, CA4, SLC5A2, ADA, HRAS, LGALS3, LGALS9, CA6, ADK, FUCA1, AKR1B1, TYMP, AKR1C3, SLC29A1, SLC5A1, IGFBP3, ADORA2B, ATIC, ALOX12, PNP, PABPC1, SLC5A4, MMP13, AMPD3, MMP1, MMP7, MMP12, MMP8	36
28	Isochlorogenic acid A	AKR1B1, MMP2, MMP12, APP, MMP13, AKR1B10, ELANE, SLC37A4, CA4, PRKCD, CA2, CA1, CA9, ABCB1, PDE9A, PDE1B, POLB, PDE5A, CASP3, ABL1, EPHA2, SRC, KDR, MAP3K9, FGFR1, AURKA, BTK, PRKCA, BACE1, MME, ECE1, HCAR2, PTGDR2, EDNRA, PIM1	35
14	7-epi-Vogeloside	LGALS3, LGALS9, ADORA1, ADORA2A, ADK, OGA, ADORA3, CA1, CA9, CA2, SLC5A2, HK2, HK1, EGFR, SLC29A1, SLC5A4, SLC5A1, AKR1B1, GBA, ADORA2B, PTGS2, GAPDH, GAA, PYGM, EDNRA, TYR, ADA, PNP, LGALS7, MAPK10, HSPA5, HSPA8, PTPN11, SLC28A2	34
21	Linalool	CA2, CA1, CA4, TRPV3, TRPM8, NR3C2, NR3C1, PGR, SLC6A3, SIGMAR1, HSD17B2, SQLE, HMOX1, IDO1, DRD2, ADRA2C, PTGS2, OPRM1, OPRD1, OPRK1, SCN5A, SCN9A, PTAFR, PARP1, ADRA1A, JAK1, JAK2, AR, MAPK8, LRRK2, TYMS, HRH3, HRH4, LTA4H	34
18	Vogeloside	LGALS3, LGALS9, ADORA1, ADORA2A, ADK, OGA, ADORA3, CA1, CA9, CA2, SLC5A2, HK2, HK1, EGFR, SLC29A1, SLC5A4, SLC5A1, AKR1B1, GBA, ADORA2B, PTGS2, GAPDH, GAA, PYGM, EDNRA, TYR, ADA, PNP, LGALS7, MAPK10, HSPA5, HSPA8, PTPN11, SLC28A2	34
15	Secologanic acid	ADORA1, ADORA2A, FUCA1, TYR, ADK, CA2, CA1, CA9, CA6, CA4, CA3, AKR1C3, LGALS3, LGALS9, ADORA3, FOLH1, AKR1B1, PNP, HK2, HK1, ADA, HRAS, SLC5A2, HSP90AA1, ATIC, ALOX12, TYMP, SLC5A1, SLC5A4	29
17	Sweroside	ADORA1, ADORA2A, FUCA1, TYR, ADK, CA2, CA1, CA9, CA6, CA4, CA3, AKR1C3, LGALS3, LGALS9, ADORA3, FOLH1, AKR1B1, PNP, HK2, HK1, ADA, HRAS, SLC5A2, HSP90AA1, ATIC, ALOX12, TYMP, SLC5A1, SLC5A4	29
10	Adinoside F	IMPDH1, MMP3, MMP9, MMP1, ADAM17, ADORA1, ADORA2A, ADORA3, ADORA2B, MMP13, MMP7, MMP12, MMP8, SLC5A2, SLC29A1, ST6GAL1, MMP2, SLC5A1, IL2, ADA, CA2, CA1, CA9, TOP1, HSPA8, DNMT1, ADK, HRAS	28
6	Luteolin-7-o-glucoside	TNF, IL2, AKR1B1, ADORA1, XDH, CA2, NOX4, ADRA2C, ALDH2, NMUR2, ADRA2A, ACHE, RPS6KA3, CA4, CD38, PRKCA, MMP1, MMP7, MMP8, CA1, CA9, ALOX5, PTGS2, SLC29A1, HSP90AA1, PLG	26
27	Neochlorogenic acid	AKR1B1, AKR1B10, MMP13, MMP2, APP, MMP12, ELANE, SLC37A4, PRKCD, PRKCA, BACE1, PDE4D, PDE9A, PDE1B, CA6, ABCB1, CA2, CA1, CA9, NEU2, CASP3, CASP6, CASP7, CASP8, CASP1	25
26	Vanillic acid	CA2, CA1, CA9, CA3, CA6, CA4, TPMT, TTR, FUT7, KDM6B, FTO, KDM4C, FYN, LCK, FBP1, AKR1C3, MMP9, MMP1, MMP2, MMP8, SQLE, POLA1, POLB, SERPINE1, TUBB1	25
23	Chlorogenic acid	AKR1B1, AKR1B10, MMP12, MMP13, MMP2, APP, ELANE, SLC37A4, PRKCD, PRKCA, CA2, CA1, CA9, BACE1, PDE4D, PDE9A, PDE1B, CA6, ABCB1, NEU2	20
25	Protocatechuic acid	CA2, CA1, CA6, CA9, CA4, CA3, FUT7, SQLE, LDHA, LDHB, TTR, ESR2, COMT, BCL2L1, IGF1R, ALK, SERPINE1, AKR1C3, GPR35, ALB	20
3	Hyperoside	NOX4, ADRA2C, AKR1B1, CA2, CA4, ACHE, RPS6KA3, NMUR2, ADRA2A, PTGS2, CD38, PDE5A, TNF, IL2, ADORA1, XDH, ALOX5, SLC29A1, TERT	19
9	Lonicerin	IL2, XDH, TNF, ADORA1, AKR1B1, NMUR2, ADRA2A, ADRA2C, ACHE, NOX4, CA2, RPS6KA3, PTGS2, CD38, PRKCA, CA4, ALDH2, PDE5A, CA1	19
2	Rutin	NMUR2, ADRA2A, ADRA2C, ACHE, AKR1B1, CA4, NOX4, CA2, RPS6KA3, XDH, CD38, PTGS2, PDE5A, TNF, IL2, ADORA1, ALOX5, TERT	18
20	Geraniol	SQLE, PTGS1, PTGS2, PGR, HMGCR, KCNH2, UGT2B7, EPHX2, JAK1, JAK2, CYP11B1, CYP11B2, PIM1, PIM3	14
12	Loganic acid	NEU2, ADORA1, SELP, SELL, CA2, CA1, CA9	7
16	Secoxyloganin	SELP, ADORA1, LGALS3, NOD2	4

**Table 2:** Top 20 targets of degree value in component-target interaction network.

Target	Degree	Target	Degree
CA2	27	MMP12	12
CA1	25	MMP13	12
CA9	22	CA3	11
CA4	20	APP	11
AKR1B1	20	TTR	10
ADORA1	19	TYR	10
CA6	15	AKR1C3	10
MMP2	14	MMP9	10
PTGS2	13	ALOX5	10
ADORA2A	13	ACHE	10
AKR1B10	12	ABCB1	10

**Table 3:** Top 20 targets of the protein-protein interaction network.

No.	Targets	Degree	Betweenness centrality	Closeness centrality
1	PIK3CA	45	0.056863	0.420118
2	MAPK1	44	0.146963	0.441909
3	PIK3R1	43	0.045671	0.418468
4	SRC	41	0.048756	0.416016
5	APP	38	0.124074	0.394444
6	HRAS	35	0.020650	0.375661
7	STAT3	34	0.097739	0.408829
8	HSP90AA1	31	0.087902	0.407266
9	NRAS	30	0.017837	0.365352
10	FYN	29	0.009133	0.381720
11	AKT1	27	0.048552	0.397388
12	EGFR	27	0.044610	0.404175
13	JAK2	27	0.011099	0.370435
14	MAPK8	27	0.031883	0.401887
15	LCK	26	0.005706	0.376991
16	PRKCD	26	0.040957	0.387273
17	PTPN11	24	0.005269	0.371080
18	NMUR2	24	0.017057	0.345779
19	JAK1	23	0.004552	0.372378
20	RELA	23	0.036432	0.401130

**Table 4:** Top 10 pathways ranked according to *P* value.

Pathway	*P* value	Count
hsa04933: AGE-RAGE signaling pathway in diabetic complications	7.94*E* − 24	23
hsa04066: HIF-1 signaling pathway	3.56*E* − 22	22
hsa01521: EGFR tyrosine kinase inhibitor resistance	1.09*E* − 21	20
hsa05205: proteoglycans in cancer	5.5*E* − 21	27
hsa04931: insulin resistance	3.31*E* − 20	21
hsa04917: prolactin signaling pathway	8.97*E* − 20	18
hsa04151: PI3K-Akt signaling pathway	5.81*E* − 19	31
hsa01522: endocrine resistance	1.8*E* − 18	19
hsa05161: hepatitis B	2.05*E* − 17	21
hsa05230: central carbon metabolism in cancer	2.22*E* − 17	16

**Table 5:** Binding energy (kcal/mol) of *Lonicerae japonicae* flos molecular docking.

No.	Component	Target			
		AKT1	PIK3CA	PIK3R1	MAPK1
C1	3-O-Methylquercetin	-4.27	-4.73	-4.17	-4.39
C15	Secologanic acid	-5.98	-4.69	-5.52	-5.24
C11	Adinoside F	-5.4	-3.73	-5.9	-6.01
C2	Apigenin	-5.48	-4.2	-5.88	-5.2
C6	Luteolin	-5.37	-3.98	-4.79	-5.78
C4	Kaempferol	-4.55	-4.23	-5.8	-5.16
C8	Quercetin	-5.54	-4.69	-4.66	-4.26
C22	Caffeic acid	-6.11	-3.91	-4.52	-4.25
C19	Alpha-terpineol	-5	-4.69	-4.2	-4.85
C29	Vanillic acid	-6.52	-4.04	-3.89	-4.07
C28	Protocatechuic acid	-6.39	-3.91	-3.93	-4.15
C24	Ferulic acid	-5.19	-3.39	-4.08	-4.8
C10	7-epi-Vogeloside	-4.54	-3.21	-3.42	-3.84
C7	Luteolin-7-o-glucoside	-3.86	-3.58	-3.74	-3.82
C21	Linalool	-4.44	-3.43	-3.31	-3.64
C23	Chlorogenic acid	-5.17	-2.82	-3	-3.68
C20	Geraniol	-4.12	-3.12	-3.67	-3.71
C27	Neochlorogenic acid	-4.94	-2.06	-2.78	-4.34
C13	Loganic acid	-4.59	-2.6	-3.48	-3.15
C17	Sweroside	-3.93	-2.72	-3.54	-3.41
C18	Vogeloside	-4.5	-3.05	-2.82	-3.22
C16	Secoxyloganin	-3.75	-1.42	-1.95	-3.7
C25	Isochlorogenic acid A	-3.72	-1.92	-3.51	-1.28
C14	Loganin	-4.12	-1.27	-1.97	-2.32
C5	Lonicerin	-1.93	-0.45	-3.76	-2.42
C12	Adinoside G	-3.69	-0.76	-1.39	-2.45
C26	Isochlorogenic acid C	-2.91	-2.34	-1.13	-1.51
C3	Hyperoside	-2.19	-0.75	-2.39	-2.24
C9	Rutin	-1.24	-0.42	-1.82	-1.19

## Data Availability

The raw/processed data required to reproduce these findings cannot be shared at this time as the data also forms part of an ongoing study.
